# Enhancing Research Involvement of Young People With Lived Expertise: Reflecting on Experiences in Digital Mental Health Research

**DOI:** 10.2196/55441

**Published:** 2024-10-18

**Authors:** Josephine Brogden, Zsofi de Haan, Carla Gorban, Samuel J Hockey, Alexis Hutcheon, Frank Iorfino, Yun Ju C Song, Elizabeth Scott, Ian B Hickie, Sarah McKenna

**Affiliations:** 1 Brain and Mind Centre The University of Sydney Camperdown, NSW Australia

**Keywords:** youth mental health, digital mental health, lived expertise, young people, youth, adolescence, technologies, university, universities, Sydney, real-world, engagement, work environment

## Abstract

Given the rapid development of digital mental health technologies and a focus on connecting with youth, there is an urgent need to enhance the engagement of young people with lived expertise in research. Even so, youth with lived experience of accessing mental health services are particularly affected by power imbalances and may receive limited compensation in academic settings. Therefore, an emphasis on how research engagement not only improves the work but can benefit young people themselves is required. Here, 5 young people with lived expertise report on their experience of being employed as researchers at the University of Sydney’s Brain and Mind Centre. As such, this team is uniquely placed to offer reflections from their work across multiple stages of research. This led to four key insights, including (1) creating accepting work cultures, (2) providing diverse opportunities for involvement, (3) giving young people agency and flexibility around sharing lived experiences, and (4) creating accommodating work environments for all researchers. We suggest that these insights can support more diverse ways of engaging young people and maximizing the value of participation for both researchers and young people themselves.

## Introduction

In Australia, 26% of young adults (aged 15 to 25 years) have experienced a long-term mental health or behavioral condition, representing a major health crisis [1]. This has prompted the rapid development of digital technology solutions, such as e-therapies and tracking tools, to provide support options that are youth-friendly and accessible. Yet, while clinical trials in controlled settings have frequently demonstrated the potential effectiveness of these technologies, these trials often report high attrition rates and decreasing usage over time [2-4]. For this reason, there are increasing calls to involve young people in digital mental health research so that new tools are optimally designed to suit the needs and preferences of the populations they seek to help [5-7].

Given this is the case, it is important to consider what young people themselves can gain from their involvement. It is concerning to find limited data regarding the value of research involvement for young people and, more particularly, how this value can be enhanced [5,8-11]. For example, a 2023 targeted review of youth involvement in mental health research found 16 articles that had described both youth involvement in research and the impacts of that involvement [12]. In work that does exist, the scope of young people’s involvement is limited as compared with other groups. The 2023 review found that youth were engaged at the advisory level in 4 studies, at the consultation level in 5 studies, at a partnership level in 8 studies, and only 1 study was “youth-led” [12]. In a more targeted review of youth involvement in digital mental health, no studies involved youth in the planning or leadership of research [13]. By contrast, a review of 70 articles detailing partnerships with adult patients in health research found that most research included patients across multiple stages, and 38 had included patients in the whole research cycle, including planning stages [11]. Thus, it is important that researchers consider more diverse types of contributions that young people with lived expertise can make, as well as the potential value of expanding their involvement.

When considering the depth of consumer involvement, Arnsteins’ ladder of participation has been hugely influential in both academic and public policy spaces. This ladder conceptualizes “citizen-control” as the highest form of participation, suggesting that projects should ideally be led by young people with lived experience of mental ill-health, including having key roles in decision-making and planning [14]. There is also an acknowledgment that this “ladder” may need to be more flexible and dynamic when working with youth populations, who often prefer to choose and change the depth of their involvement based on their interests [15]. Hence, while there is a need to increase young people’s involvement in decision-making and leadership roles, young people should have a say in designing their involvement and may not always want “citizen control.” Ultimately, it is incumbent upon research institutions to create optimal conditions for young people to have agency and flexibility regarding their own involvement [5,16]. In addition, much attention has been given to how researchers interact with individuals with lived expertise, not just what activities they are involved in. While frameworks are often project-specific, key principles that are continuously promoted include supportive institutional policies, supportive attitudes that ensure strong communication and shared goals, mutual respect, addressing training needs, providing resources and advanced planning, and recognizing the value of partnership throughout all stages of research [5,16].

Taken together, while increasing engagement between young people and researchers is vital, we should also consider the extent to which this participation is benefitting young people and whether key principles of involvement are being realized, in other words, addressing whether young people do feel respected and recognized by academic researchers. Young people themselves should be supported in providing their own reflections in their own words. There are increasing calls to elevate lived expertise perspectives of being involved in research, given a longstanding history of coercion and damaging power dynamics in academic settings [17,18]. When planning research involvement of individuals with lived expertise, it is important for academic researchers to be empathic and understanding about how this involvement will be experienced, particularly by vulnerable populations. Furthermore, they should seek to enhance the benefits of research participation for these individuals where possible.

The Youth Mental Health and Technology team at The University of Sydney’s Brain and Mind Centre collaborates on a range of projects related to improving youth mental health services, partly through novel digital tools. Our team has consistently documented involving young people with lived expertise in our work [19-21]; however, our partnerships with young people were strengthened in 2021 by the establishment of a lived experience working group, coled by permanent lived experience researchers on our team, and documented in a previous publication [22]. In addition, our team has successfully employed 5 young people as lived expertise researchers on an ongoing basis to ensure that young people are given more flexibility and autonomy in their contributions to our work. This has also led to stronger collaborations between academic researchers and young people in our community more broadly. Accordingly, 5 lived expertise researchers on our team wrote independent reflections and participated in reflective group discussions. We hope that this can provide a greater understanding of how to enhance the value of research involvement for young people themselves in line with existing frameworks and guidelines.

## Overview of Lived Expertise Research Team and Reflective Process

### Ethical Considerations

Our team has published a framework that underpins our team’s lived expertise contributions to research [22] and has received ethical approval from the University of Sydney’s Human Research and Ethics Committee (Project number: 2020/786) for this work. Given that lived expertise researchers are reflecting on their own experiences, we did not seek informed consent. Even so, quotes and reflections were deidentified so that lived expertise researchers could provide commentary confidentially. All lived expertise researchers were hired as casual, full-time, or part-time professional staff.

### Description of Lived Expertise Research Team and Responsibilities

In this paper, the term “lived expertise researcher” refers to young people with lived experiences of seeking treatment for mental ill-health. In line with Killackey’s framework, most lived expertise researchers also had other types of expertise in mental health, including laboring (which includes working in mental health services), loving (caring for someone with mental ill-health), and learning (being involved in mental health research) [23]. By contrast, this paper will refer to all academic staff with learned expertise and no declared lived expertise in mental ill-health as “academic researchers.” The lived expertise researchers on our team are between 20 and 31 years old and have been employed for between 9 months and 5 years. They have been involved in a range of projects at various stages. Furthermore, they have moved between advisor, partner, and decision-making roles [22,24,25]. Table 1 describes the research activities of this team according to the Smits et al [24] involvement matrix. Many of these roles have been described in more detail in previous publications [22,25,26].

**Table 1:** Overview of lived expertise researchers’ involvement in projects conducted by the Youth Mental Health and Technology team.

Role in project	Nature of lived expertise researchers’ involvement in project	Stage of project when this occurred
Listener is given information	Grant funding needed to be sought before hiring lived expertise researchers, so the major responsibilities of these roles had been designed before lived expertise researchers joined the team.	Preparation^a^
Cothinker is asked to give opinion	Lived expertise researchers are included in regular team meetings during which new projects or grant proposals are being discussed, researchers provide updates on ongoing projects and troubleshoot problems, and findings from data analysis are discussed and interpreted.	Preparation Execution^b^ Implementation^c^
Advisor gives unsolicited advice	Lived expertise researchers work alongside academic researchers as part of working groups that are developing new digital tools and helping to design user flow and content.	Preparation Execution Implementation
Partner works as an equal partner.	A lived expertise researcher has contributed to designing procedures for an ongoing clinical trial, as part of which they perform the role of “Digital Navigator,” assisting young people to understand how to use a novel digital tool. This included partnering with the academic lead to design technology implementation protocols, role descriptions, and qualitative data collection tools. This lived expertise researcher has also assisted with recruitment and data collection and is a co–first author on publications about the novel role that they have performed in this trial. Lived expertise researchers have coled the development and ongoing running of a lived expertise working group and have coled qualitative workshops. This has included creating protocols, assisting with ethics proposals, recruiting members, organizing meetings, collecting evaluative data, and contributing to outputs.	Preparation Execution Implementation
Decision maker takes initiative (final) decisions	A lived expertise researcher has developed an information workshop to translate the Brain and Mind Centres Model of Care (that underpins the digital tools we are developing) to young people. This will be delivered to health services and evaluated.	Implementation

^a^Preparation stage includes developing ideas, formulating questions, financing, and ethical approval.

^b^Execution stage includes recruiting study subjects, choosing instruments, data collection, and data analysis.

^c^Implementation stage includes report writing, dissemination in the media, and translation to practice.

### Reflective Process and Approach to Data Analysis

To ensure that all members of our team had an equal opportunity to contribute, despite varying experience and training in academia, we used a process of coreflection that was designed by our team and was underpinned by the principles of thematic analysis [17,27] as this approach allows both inductive and theory-driven data analysis. Our approach followed reflective processes that had previously been adopted by lived expertise groups to provide their collective experiences of being involved in research [17]. Based on existing guidelines, a clinical researcher was also involved in providing guidance and support, as this can improve young people’s level of involvement in academic projects [15].

Young people wrote personal reflections using the prompt, “How have you experienced being a member of the Lived Expertise team?” This broad prompt allowed us to identify key aspects of the research experience from the perspective of our team rather than being encouraged to focus on key topics. Following this, all team members were given several weeks to read over the reflections, which informed 2 group discussions, both of which were held over 2 hours through Zoom (Zoom Video Communications), the focus of which was to identify common topics. Discussions were facilitated by a clinical researcher, video recorded, and later transcribed. Subsequently, a clinical researcher (SM) who was trained in thematic analysis and a lived expertise researcher (SJH) summarized topics into themes based on these discussions and existing frameworks on participatory practices. All lived expertise researchers also contributed to the drafting of this paper to ensure it reflected their beliefs and wishes. For example, the first draft of this paper was edited so that language use was more inclusive.

Data analysis was informed by thematic analyses, as themes were derived from what was in the data (written reflections and discussions), as well as from existing frameworks on creating supportive partnerships between academic and lived expertise researchers [5,27]. This was done to ensure that we captured themes that were most relevant to the lived expertise researchers themselves while also considering existing literature on creating positive experiences for young people. Data analyses followed the constructivist grounded theory that argues all knowledge is constructed by meanings that individuals bring to data analysis [28]. As this was a group reflective process, researchers’ own perspectives and experiences influenced the organization of data into themes and categories.

## Insights From Lived Expertise Researchers on a Digital Mental Health Team

### Creating an Accepting Culture and Addressing Academic Ableism

Academic institutions are often seen as ableist and exclusionary by those with lived expertise, particularly young people, due to unequal compensation and opportunities for development as compared with academic staff. Previous reviews have found that the risks of hiring researchers with disabilities are overemphasized as compared with the benefits, that lived experience is not valued in the process of fellowship schemes, and that, in academic institutions, there are few individuals with disabilities of any kind in positions of power [29]. As such, there is a need to enable greater inclusion in academic institutions.

There was an uncomfortable feeling of being exposed before I had even arrived... I was worried about how I would navigate this awareness with my colleagues, but my apprehensions were allayed almost instantly. The Digital Health team welcomed me as a researcher first, with valuable, unique insights and able to contribute to the research projects, not solely because of my lived experience.

For this lived expertise researcher, it was rewarding to be valued because of experiences that are generally highly stigmatized, and being so highly valued by colleagues also led to a sense of belonging and acceptance within their workplace. A lived expertise researcher who had been on the team for over 5 years felt that this increased acceptance was linked to a change in culture over time.

There has been a necessary encouragement toward an overall culture change in the role and importance of lived experience. I believe this to be from a new understanding of the process of inclusion in academic outputs... One main problem is ableism, one of the core things for me has been the differences in pay, and the fact that you can’t go beyond a certain point if you don’t have an academic qualifications.

Relatedly, other lived expertise researchers felt that because of “ableist” policies in academic institutions, it is important for research teams to actively build a culture of inclusivity and acceptance.

In a broader research team, there's maybe less of an understanding about where the value comes from as someone with lived experience, and then maybe more of a sense of a need to justify that by sharing some of your lived experience.

Sometimes it is hard to recognise what I bring to the table in a team of very accomplished researchers. So while no one has actively said or done anything to me I think the academic environment sometimes reinforces this insecurity. Being included as a core member of our team has been an important step in recognising that my lived experience adds value and is not something I need to use to justify why I am here.

Overall, cultural and systemic problems in academic institutions can create power imbalances that negatively impact lived expertise researchers. Yet, when academic researchers can create welcoming and supportive environments for those who have traditionally been excluded from research, this can be highly rewarding for individuals. Furthermore, this can lead to more accepting workplace environments that benefit all researchers.

### Providing Diverse Opportunities to Use a Variety of Skills

Given the nature of our teams’ work, which is focused on designing and implementing new digital tools, lived expertise researchers were encouraged to contribute to projects in a variety of ways as shown in Table 1. Furthermore, these opportunities were not always linked to their lived expertise but allowed them to build capacities in new areas or to use other existing skill sets. For example, lived expertise researchers who were originally employed to implement a specific digital platform in services have also been involved in the planning and development of further digital tools, partly due to their experience working in health services or their clinical training.

The really great part about working in a broader research team is that you can actually be valued for other skill sets, and other parts of who you are… like, you know that you're also intelligent, or you're really good at writing or whatever it may be. There are other things that make you who you are, that are outside of this mental illness that's defined your life in many ways.

[I have been] given opportunities, that I wouldn't necessarily think that I was capable of doing, [and wouldn’t have] put my hand up for… [My manager] doesn't make assumptions. She's like, you don't have to but yeah, and it's that exposure therapy thing. That's really helped me build up confidence as hard as it is.

Importantly, all lived expertise researchers on our team agreed that lived expertise advisory groups were highly valuable and rewarding in and of themselves, yet having the opportunity to contribute more diverse skills significantly enhanced the value of research involvement. As expressed by one lived expertise researcher below:

I think something that I'm like cautious of [is that] oh, are [academic researchers] gonna, like see me as someone who's lived experience and now assume that there's a set of things I can and can't do… you’re just like this young lived experience person, is there anything else more than that? And the answer is like always, yes.

Taken together, academic researchers can enhance the value of being involved in research by creating diverse opportunities for young people with lived expertise. Given the rapid progress being made in digital mental health, this field is particularly likely to benefit from more reflexive involvement of individuals with diverse skill sets.

### Creating Agency and Flexibility Around Sharing Lived Experiences

Again, it was rewarding for our lived expertise researchers to be in an environment where their experiences of mental ill-health were valued, given that these illnesses continue to be highly stigmatized. [5,30] At the same time, as expressed by one lived expertise researcher below, publicly declaring mental ill-health was a difficult decision and did come with challenges.

Not having the luxury of hiding my mental-ill health at work has left me feeling vulnerable at times... Initially, I thought I had to reveal everything about myself so that others would consider my experiences valid enough. Despite having an on-going battle with this, the team's encouragement and inclusive culture minimise my insecurities that I am not accomplished or sick enough to be called a lived experience researcher.”

Thus, we believe it is important for all academic colleagues involved in mental health research to have training and additional support regarding how to openly and safely discuss experiences of mental ill-health. Without this step, lived expertise involvement may be limited, and the value may not be realized because of academic researchers’ own attitudes and stigmas. For example, a lived expertise researcher who was originally employed to assist with the implementation of a technology platform in health services has also been involved in the development of further digital tools. This researcher reflected on an interaction they had had with other researchers on the project, who appeared uncomfortable when she discussed her lived experience.

My experience so far working with other researchers in the team and other projects that I'm on, is they get a real shock if I do reference my lived experience, and they get really uncomfortable about it… I was curious to observe that my colleagues initially did not know how to respond to me when I shared parts of my lived experience related to our project. Further, that they felt they needed to acknowledge my value as coming from my clinical experience, rather than my lived experience. Almost as though they wanted me to see I was more than my lived experience. In time this team has responded very positively when I have shared lived experience and go out of their way to seek my lived experience input now they know me more.

It is also important that lived expertise researchers are fully empowered to choose how, when, and what they share of their personal experiences. One individual appreciated that academic researchers supported her in choosing what and when to share.

We’ve all had parts in our lives and different jobs where we've had to hide it… And I think it's, it's unusual for me to actually have a group of people that encourage you to sort of let go a little bit, and I struggle to, to let my walls down… [But] it's also being really respected about what I share and don't share… I've been able to sort of weave my lived experience and mental illness into the conversations in a way that I'm comfortable.

Therefore, academic researchers may need more support to discuss mental ill-health openly and safely and must understand how to give lived expertise researchers agency and flexibility around sharing their personal stories.

### Creating More Accommodating Workplaces for All Researchers

Finally, these discussions caused our lived expertise researchers to reflect on the need for research institutions to become more accommodating workplaces generally. Provisions such as more flexible working arrangements, as well as increased training and support, helped our team to successfully contribute to our research and have meaningful work experiences. Yet, these accommodations should be made more routine across academic workplaces (and all other workplaces) to ensure that research benefits from a diverse range of voices with all types of backgrounds and skills. In addition, 2 lived expertise researchers provided reflections on how workplace accommodations had helped them to succeed.

Navigating work responsibilities, medical appointments and keeping on top of my mental health can be overwhelming and has impacted my performance at times. Having a manager who supports me, allows the flexibility to work from home, attend mental health appointments and provides a safe space to talk openly has been crucial for my success in this role.

I currently have a flexible working arrangement, working between 5 – 10 hours per week on a casual basis. I feel incredibly supported with my workload and am able to work within my capacity. This allows me to maintain a strong work life balance, manage my mental and physical health, as well as pursue my studies. I also have frequent supervision meetings, as well as peer support meetings, which allow me to properly manage my workload, and learn from other lived experience team members.

In conclusion, our experiences demonstrate that it is not only feasible for research institutions to make better workplace accommodations but also an important step to ensuring that the skills and experiences of all individuals from all backgrounds are being used to their full potential.

## Summary

Taken together, this paper summarizes our experiences of integrating 5 young people with lived expertise in mental ill-health across a range of digital mental health research projects. These steps include (1) creating accepting work cultures and addressing academic ableism, (2) providing diverse opportunities for involvement, (3) giving young people agency and flexibility around when and how to share lived experiences, and (4) creating accommodating work environments for all researchers. These reflections have important implications for youth mental health research, as they demonstrate key steps that academic researchers can take to enhance the value of lived expertise research, particularly for the young people involved. Textbox 1 provides a summary of why such reflections are important from the perspective of one of our lived experienced researchers.

**Commentary on findings from a lived expertise researcher (SJH).**

Our paper highlights that cultural and systemic changes are necessary for research institutions to improve inclusivity and safety for all individuals. The current definition of a “researcher” is someone from science, academia, or clinical disciplines. Inherently, these disciplines come from a knowledge or expertise rooted in education and training. Comparatively, our understanding of the research subject—the “researched”—is someone having lived and living experience of mental ill-health. In their role, lived, and living experience, individuals are solely there to share their experiences on a consultation or advisory basis.

Equal to knowledge-based expertise, individuals with lived and living experience have lived experience-based expertise that pertains to mental health. Therefore, a new understanding—redefinition—is needed to acknowledge the value that individuals with lived and living experiences hold as researchers and not just the researched, or paradoxically in some cases, a combination of both. To address this change in researcher credentials, clarity of roles, expectations, and role extent is needed.

A key reason for this change is that prejudices, pay inequities, oversight of qualifications, and dismissal of lived expertise researchers ultimately cap career progression for many. The previously mentioned definitions of a researcher have shaped a surreptitious culture of academic bias and confusion to value, expectations of experience remit, and enrichment of research lived-expertise researchers bring [7]. Commonly lived expertise researchers experience dismissal, inadequacy, and lack of qualifications due to this bias and lack of clarity, often oversharing at their own expense to vie for their worth [7,16]. Sharing one’s lived and living experience daily is emotionally burdensome; without modification of the current culture, this potential of oversharing can impact a lived expertise researcher’s mental wellness [7,31].

Institutionally, this must change. Due to the size of academic institutions (like universities), a combined approach of top-down and bottom-up would be needed for this to happen. An example of implementable, bottom-up approaches is that responsibilities held by supervisors and academic staff foster the current culture and, consequently, are accountable for social and systemic academic ableism. By facilitating opportunities to upskill and build research skill sets, supervisors enable further career progression for lived expertise researchers due to increased abilities and responsibilities.

A key strength of this work is that young people have been able to compare and contrast the benefits and disadvantages of different types of engagement and, therefore, to provide important recommendations about how to enhance the benefits and mitigate the risks of lived expertise involvement in youth mental health research. While much has been written about the impacts of lived expertise involvement, recent meta-analyses found that young people were underrepresented in research as, unlike adult populations, they are generally not involved in planning stages, and their involvement is often restricted to working groups or advisory panels [11,13]. Generally, academic researchers have sole decision-making power when identifying how to enhance the value of involving young people in research. Meanwhile, young people themselves lack opportunities to critically reflect on ways to improve the value of their engagement. By contrast, this paper included perspectives from 5 young people with lived expertise in mental ill-health who have been involved in a range of different projects at different stages and in a variety of ways. As such, a key contribution to our work is demonstrating how young people can be integrated into a research team and drive more diverse and valuable contributions to research in partnership with academic researchers.

Despite these contributions, our paper has important limitations. This piece reflects our teams’ personal perspectives of being involved in digital mental health research and is based on the experiences of 5 individuals. We believe that highlighting the perspectives of individuals with lived expertise is an important step toward improving the accessibility of academic research. However, this approach and limited sample size also impact the generalizability of our reflections. Furthermore, it is important to acknowledge that lived expertise researchers are still employed by our team, and there are inherent risks involved in reflecting openly on their experiences, which may also have biased the perspectives they were willing to provide. As such, we took several steps to mitigate these risks. First, we deliberately designed a group reflective process so that individual researchers were able to share their perspectives without needing to identify themselves and their opinions to academic researchers on the team; all quotes used in this piece are anonymous for the same reason. Second, we have included leading academic researchers as coauthors to show that the perspectives of our lived expertise researchers, including critical perspectives, are fully supported and endorsed by our team. Third, existing guidelines suggest that vulnerable populations may need additional support to increase their level of involvement in research [15,32]. In line with these recommendations, a clinical researcher, who was a clinical psychologist and academic researcher, was involved throughout all stages of planning, data collection, analysis, and write-up to provide support as needed. Finally, we have sought and been given ethics approval by the relevant university human ethics committee to ensure that young people’s involvement in our research is conducted safely.

In conclusion, the benefits of being involved in research for our team were that they felt valued for their experiences rather than stigmatized, they felt accepted by academic researchers, they were able to contribute a variety of skills and to develop others, and they were given accommodations when needed to maintain a healthy work-life balance. This, in turn, highlights important steps that researchers can take to ensure that academic work and young people themselves are benefitting fully from lived expertise involvement in research.
